# A Prospective, Noninterventional Study to Evaluate the Impact of Emicizumab in the Management of Hemophilia A

**DOI:** 10.7759/cureus.74948

**Published:** 2024-12-02

**Authors:** Anupam Dutta, Taniya S Dutta, Luish Borbouah, Yash Duseja, Juhi Bora, Papori Gogoi

**Affiliations:** 1 Department of General Medicine, Assam Medical College and Hospital, Dibrugarh, IND; 2 Department of Pediatrics, Assam Medical College and Hospital, Dibrugarh, IND; 3 Department of Medicine, Assam Medical College and Hospital, Dibrugarh, IND

**Keywords:** bleeding rate, coagulation factor viii, emicizumab, functional independence, hemophilia a, quality of life, visual analog score

## Abstract

Background and objective

Hemophilia A (HA) is a genetic bleeding disorder caused by a lack of factor VIII (FVIII) and is associated with frequent bleeding and joint damage. Traditional intravenous treatments for this condition are cumbersome and can lead to complications. Emicizumab, a bispecific monoclonal antibody, offers a promising subcutaneous alternative with potential safety and efficacy-related benefits. This study aimed to evaluate the impact of emicizumab prophylaxis on bleeding rates, joint health, functional activity, and quality of life (QoL) in patients with congenital HA.

Methods

A noninterventional, prospective observational study was conducted at the Assam Medical College, a tertiary care center in northeastern India, involving 40 patients with HA (PwHA), who were either on FVIII therapy or newly on emicizumab. Emicizumab was given subcutaneously at 3 mg/kg weekly for the first month, followed by 6 mg/kg every four weeks. Endpoints included changes in annual bleeding rate (ABR), Hemophilia Joint Health Score (HJHS), Functional Independence Score in Hemophilia (FISH), and QoL via European Quality of Life 5-Dimensions 5-Levels (EQ-5D-5L) and visual analog scale (VAS) scores at 24 weeks.

Results

At 24 weeks, HJHS improved from 12.8 to 4.8 (p<0.001), FISH from 27.5 to 30.6 (p<0.001), and ABR decreased from 11.36 to 0.195. Quality of life scores also improved (EQ-5D-5L index from 0.79 to 0.96, VAS from 72.18 to 92.75, both p<0.001). All 30 patients with target joints had resolved bleeds, and adherence to emicizumab was 100%.

Conclusions

Based on our findings, emicizumab significantly reduces bleeding, enhances joint health, and improves the quality of life in PwHA. It is associated with high adherence, suggesting its feasibility as a treatment, especially in resource-limited settings. However, long-term studies are needed to validate these results.

## Introduction

Hemophilia A (HA) and hemophilia B (HB) are inherited bleeding disorders resulting from partial or complete deficiencies in coagulation factors VIII and IX, respectively [1]. As per the World Federation of Hemophilia (WFH) 2020 report, these conditions collectively occur at birth with a prevalence of 1 in 3333 [2]. According to the 2023 global survey by the WFH, a total of 218,804 cases of HA, HB, and cases of unknown type were reported across 119 countries [3]. Additionally, data from the World Bleeding Disorders Registry (WBDR) indicates that the highest proportion of cases (32.6%) were reported from Southeast Asia [4]. In 2020, the Indian Hemophilia Registry listed nearly 20,000 patients; this number is expected to reach 85,000-‍100,000 with the expansion of coagulation laboratories nationwide [5].

HA varies from mild to severe, depending on endogenous levels of factor VIII (FVIII). Severe HA can cause frequent spontaneous bleeding into joints or muscles, which may result in pain, swelling, arthropathy, and life-threatening conditions such as intracranial hemorrhage. Bleeding events may also lead to occupational impairment and progressive loss of mobility [6]. Long-term spontaneous joint bleeding and soft tissue issues can significantly affect a patient’s health-related quality of life (HRQoL) [7,8]. An observational study from India in 2019 reported that 65.7% of the total patients had severe HA; 69.4% of them were under 18 years of age and 63.8% were over 18 years of age, with FVIII levels below 1%. Moderate hemophilia was found in 21.3% of cases, and mild deficiency was seen in 12.9% of cases [9].

Therapy for HA is associated with two main approaches: on-demand treatment to stop bleeding when it occurs and prophylaxis to prevent bleeding. Prophylaxis is based on the correlation between bleeding phenotype and FVIII activity. Both approaches have used two therapeutic options for decades: FVIII products and bypassing agents (BPAs) [10]. Intravenous (IV) clotting factor infusions two to three times per week are necessary for prophylactic bleed prevention in HA; however, by adulthood, less than 50% adhere to treatment, leading to pain, joint degeneration, and disability [11]. WFH recommends global prophylactic treatment for HA, with less intensive options in resource-limited settings. Prophylaxis is more effective than on-demand therapy in reducing annual bleeding rates (ABRs), disability, and financial costs [12]. FVIII replacement therapy is complicated by inhibitors (neutralizing alloantibodies) in about 30% of severe HA cases, often developing early in life. This necessitates costly and sometimes ineffective use of BPAs to induce immunological tolerance [13]. In resource-rich countries, high-dose FVIII prophylaxis (25-40 IU/kg every 48 hours) is standard for HA. In low- and middle-income countries, a cost-effective low-dose FVIII (10-15 IU/kg twice a week) is commonly used [14].

Emicizumab (Hemlibra®), a humanized bispecific recombinant monoclonal antibody, acts like FVIII by binding substrate factor X and enzyme factor IXa. It is the first nonfactor medication administered subcutaneously for patients with or without FVIII inhibitors, significantly improving quality of life by reducing bleeding episodes [15]. Approved by the Food and Drug Administration (FDA) in 2017 for patients with inhibitors and in 2018 for those without, emicizumab effectively prevents treated bleeds and resolves target joint bleeds [14].

The healthcare system in northeastern India caters to a population residing in remote regions, where access to hemophilia care remains elusive. As per the limited literature on the prevalence of HA in the northeastern states of India, the burden identified reflects the unmet need for hemophilia care in this region [16,17]. The introduction of emicizumab is expected to reduce the frequency of hospital visits while achieving clinical outcomes comparable with FVIII concentrates, including diminished bleeding episodes and joint-related complications. Also, its subcutaneous application facilitates the possibility of self-administration with appropriate professional training, holding promise for the future implementation of home-based management for patients with hemophilia [15]. In India, emicizumab has been available since April 2019 for managing HA in individuals with FVIII inhibitors, though its high cost limits widespread adoption [18]. Low-dose emicizumab offers a cost-effective alternative with superior efficacy and safety compared with low-dose FVIII prophylaxis [12].

Prophylactic emicizumab should be prioritized in resource-limited settings for pediatric patients, infants with IV access challenges, patients with FVIII inhibitors, those with high ABR, and individuals with histories of life-threatening events or those living far from healthcare centers [12]. Long-term studies on emicizumab in India are rare, and only anecdotal evidence of its effectiveness is currently available [12]. At present, advanced treatments for hemophilia are confined to specialized tertiary centers. Our objective was to enhance the accessibility of these treatments and alleviate the burden on patients. Hence, this study aimed to assess the impact of initiating nonfactor-based therapy with emicizumab on bleeding rates, joint health, functional activity, and QoL in patients with congenital HA. The objective was to evaluate the efficacy of emicizumab, as compared with previous FVIII concentrates, by measuring changes in several endpoints: ABR, annual joint bleed rate, annual target joint bleed rate, joint health using the Hemophilia Joint Health Score (HJHS), functional activity through the Functional Independence Score in Hemophilia (FISH), and QoL via the European Quality of Life 5‐Dimensions 5‐levels (EQ‐5D‐5L) questionnaire and visual analog scale (EQ‐VAS).

## Materials and methods

Study setting

This hospital-based, noninterventional observational study was conducted at the Department of General Medicine, Assam Medical College and Hospital, northeastern India, between October 19, 2023, and June 19, 2024. The site had recorded patient details available from October 19, 2023, to November 19, 2023, allowing one month for recruitment and data collection from all participants. The follow-up period continued until June 19, 2024, spanning a total of six months.

Population

Given the limited number of patients with HA (PwHA), the sample size for this study was based on clinical rather than statistical considerations. Convenience sampling was performed to include 40 patients in the study. The purpose of the study was explained, and informed consent was obtained from patients or patients in vernacular language. For adolescents aged seven years and above, assent was also obtained. Patients already on emicizumab were observed and studied, with the study beginning within weeks of drug initiation. This approach ensured that the data captured reflected their QoL and bleed rates during the previous prophylactic treatment. Participants were selected based on the specified inclusion and exclusion criteria (Table 1).

**Table 1:** Eligibility criteria for the selection of study participants FVIII: factor VIII; FISH: Functional Independence Score in Hemophilia; HA: Hemophilia A; HJHS: Hemophilia Joint Health Score

Criteria	Details
Inclusion	Patients with congenital HA on regular/episodic FVIII replacement therapy with and without inhibitors
	Patients who had initiated emicizumab a maximum of three months before enrollment into the study or who initiated emicizumab treatment at enrollment, where the decision to initiate emicizumab has been made before the decision for study participation
	Patients with congenital hemophilia with high HJHS and FISH
	Patients with adequate renal and hepatic function
	Patients with adequate hematological function
Exclusion	Patients with bleeding disorders other than congenital HA
	Patients with a prior history of thromboembolic episodes
	Patients with a prior history of injection site reactions
	Patients with a history of hypersensitivity to monoclonal antibodies
	Patients with immunocompromised status

Data collection

Demographic data and medical history, particularly regarding hemophilia, were collected. A thorough examination was conducted, and any abnormalities detected at baseline were reevaluated in subsequent assessments.

Ethical considerations

This study received ethical clearance from the Institutional Ethics Committee of Assam Medical College and Hospital (Human) before starting the research. The EC approval number for the study is 2023/AMC/EC/10795 (received on October 19, 2023). The study adhered to the principles of the Declaration of Helsinki. The study's purpose was clearly communicated, and informed consent was obtained from patients or their parents in the local vernacular language. Additionally, assent was secured from adolescents aged seven years and older. Participant data were anonymized and securely stored to ensure confidentiality, in compliance with ethical standards and applicable regulations. Only authorized study personnel had access to the data, which were used exclusively for research purposes.

Methodology

Patients who fulfilled the inclusion criteria were recruited into the study after they were screened upon their visit to the hemophilia outpatients department. The patients enrolled in the study were previously on episodic/prophylactic FVIII-based replacement therapy.

*Treatment* *Drug*

Emicizumab.

*Dosing Regimen* 

3 mg/kg/week for the first month followed by 6 mg/kg once every four weeks. 

Route of Administration

Subcutaneous

Dosage Administration

The initial four doses for the first month were administered by the healthcare provider in the hemophilia clinic. Patients were observed for 60 minutes after administration. The clinicians were guided on how to administer the medication and informed regarding the correct dosage as per the instruction manual. During these first four visits, patients and caregivers were educated about potential adverse reactions to emicizumab through explanations and pictorial representations. They were also trained in drug administration. Home administration was allowed only if the patient/caregiver was able to successfully administer two doses per visit under observation. Those who preferred healthcare provider administration could continue this practice.

Dosing Schedules

**Figure 1:**
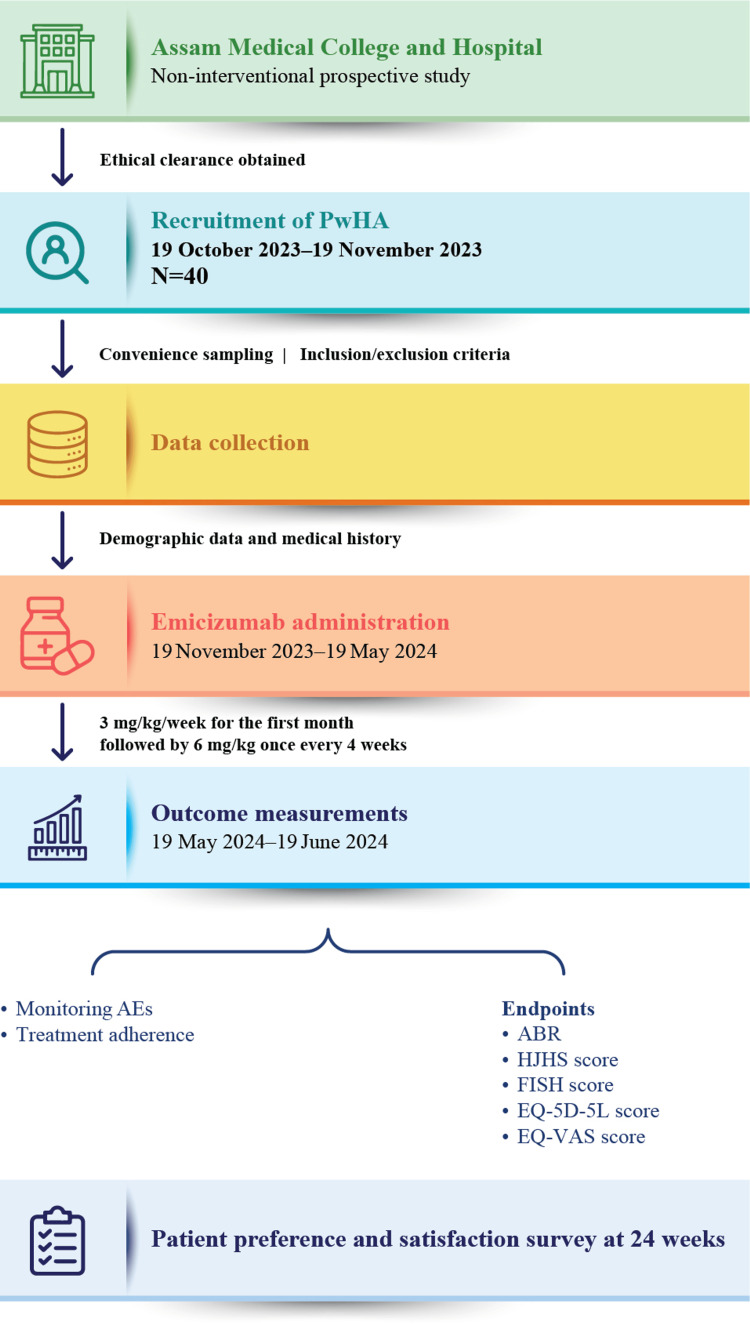
Schematic flow diagram illustrating the study methodology ABR: annual bleeding rate; AE: adverse event; EQ-5D-5L: European Quality of Life 5‐Dimensions 5‐levels; EQ-VAS: European Quality Visual Analog Scale; FISH: Functional Independence Score in Hemophilia; HJHS: Hemophilia Joint Health Score; PwHA: people with hemophilia A Image credit: Anupam Dutta

The doses were administered according to the dosing schedule prepared by the hemophilia clinic (Figure 1).

Missed Doses

For patients with once-weekly dosing, the missed dose had to be taken within three days, and those with fortnightly dosing had to take the missed dose within seven days. People who missed their doses were contacted by their phone number recorded in our registry. Patients on home management by self-‍administration were required to update the weekly/fortnightly dosing in the schedule form provided by the hemophilia clinic. These patients also had to visit the hemophilia clinic once a month to record treatment adherence and undergo a musculoskeletal examination.

Means of Communication

Patients and caregivers could ask any drug-related questions at any clinic visit. They were provided with the contact information of the hemophilia clinic for any queries related to drug dosage or adverse reactions.

Other Drugs

Patients were advised to avoid anticoagulants and antiplatelets. If these drugs were highly indicated, the specific case was discussed with the medical monitor and managed accordingly.

Dose Adjustments

If any dose adjustment was required for a patient, it was discussed with the medical monitor.

Outcome Measures

The outcomes for patients on emicizumab were measured by the following endpoints: change in joint health (HJHS and ABR); change in functional activity (FISH score); change in the quality-of-life score (EQ-5D-5L and EQ-VAS score). These endpoints were assessed 24 weeks following the initiation of emicizumab therapy. The secondary objective was to estimate the proportion of patients adhering to the nonfactor-based prophylaxis regimen. Scores for joint health, functional activity, and quality of life were assessed at baseline and 24 weeks. Consideration had to be made for patient visit dates and availability, and thus may not have been accurate, but an approximate six-month difference was maintained between the two observations.

The physicians and physiotherapists evaluated joint health, functional activity, and QoL in patients using standardized tools and a structured approach. Joint health was assessed through the HJHS, which evaluates joint function and damage, focusing on the knees, ankles, and elbows. Physicians also calculated the ABR to monitor bleeding episodes requiring intervention. Functional activity was measured using the FISH, a performance-based tool that assesses patients' ability to perform daily tasks such as walking, grooming, and running. QoL was evaluated through the EQ-5D-5L questionnaire, capturing dimensions like mobility, self-care, and pain, along with the EQ-VAS score, a patient-rated health scale from 0 to 100. These assessments were performed to track changes and ensure accurate evaluation. Physicians were also responsible for educating patients and caregivers about treatment, monitoring adherence, and managing potential adverse effects, ensuring a comprehensive approach to patient care.

HJHS: This tool was first developed in 2003. It is a physical examination tool designed to assess joint health in children with hemophilia who are aged 4-18 years. Sensitive to early signs of joint damage, it focuses on commonly affected joints-knees, ankles, and elbows and is suitable for monitoring joint changes over time or evaluating treatment efficacy in those receiving prophylactic or on-demand therapy. It is particularly useful for mild joint impairments and can guide orthopedic interventions or measure outcomes of physiotherapy treatments. It measures joint health in knees, ankles, and elbows, ranging from 0 to 124. The HJHS scales demonstrate strong construct validity, as evidenced by their moderate correlation with the physician's arthropathy impact score, individual joint score, and total score. The HJHS takes about 90 minutes to administer, though less time may be required with experience [19]. The physical examination was undertaken by physiotherapists (Figure in the Appendices).

ABR: ABR was calculated by annualizing the number of bleeds requiring treatment with coagulation factors for the six months before and after emicizumab administration.

FISH: This tool was first developed in 2005. It is a performance-based tool that objectively measures functional ability and tracks changes over time or after interventions. It complements clinical and radiological scores and is suitable for diverse linguistic backgrounds. It was developed with input from patients, relatives, and therapists FISH focuses on daily activities impacted by hemophilia, excluding unsafe or clinically unassessable tasks. It evaluates eight activities - eating, grooming, dressing, chair transfer, squatting, walking, step climbing, and running - by using clearly defined independence levels to ensure consistency. FISH is scored based on the observed performance of tasks, with each activity graded from 1 to 4 depending on the assistance needed (maximum score: 32). It takes 12-15 minutes to administer once the evaluator is familiar with the tool. Validity studies show reasonable construct validity, good face and content validity, and strong criterion validity. The tool demonstrates high reliability and sensitivity to changes in treatment [20]. It evaluated activities performed by the patient, with scores ranging from 1 to 4 (Figure in the Appendices).

EQ‐5D‐5L questionnaire: The 5-level EQ-5D version (EQ-5D-5L) consists of two components: the EQ-5D descriptive system and the EQ VAS. The descriptive system evaluates five dimensions of health: mobility, self-care, usual activities, pain/discomfort, and anxiety/depression. Each dimension has five levels of severity: no problems, slight problems, moderate problems, severe problems, and extreme problems. Patients select the statement that best represents their health state in each dimension, resulting in a one-digit score per dimension. These digits are combined into a five-digit code that represents the patient's overall health state [21]. Thus, this questionnaire assesses five different dimensions related to different aspects of life and grades each dimension at five levels (Table in the Appendices).

EQ‐VAS Score: It captures a patient’s self-rated health on a vertical scale, ranging from "The best health you can imagine" to "The worst health you can imagine." This provides a quantitative measure of health outcomes based on the patient’s subjective judgment [21]. The score ranges from 0 to 100 (Figure in the Appendices).

Validated questionnaires were used to measure these outcomes (Figures and the Table in the Appendices).

Patient Preference and Satisfaction

Patient preference and satisfaction with emicizumab treatment were assessed at 24 weeks through the preference survey. The survey captured the treatment option that was received better by patients treated with IV FVIII (episodic or prophylaxis) or subcutaneous emicizumab.

Safety Assessment

Participants were regularly monitored for the development of systemic hypersensitivity, anaphylaxis, and other adverse events (AEs) such as arthralgia, injection site reactions, and headaches. Aspects such as incidence and grading of AEs, thromboembolic events, severity of injection site reactions, AEs leading to discontinuation of the drug concerned, severe hypersensitivity, and occurrence of anaphylactic reactions were closely recorded. Figure 1 illustrates the study’s methodology, detailing key steps from patient recruitment to data analysis, including treatment administration, monitoring, and outcome assessments.

Statistical Analyses

The data were analyzed using MS Excel Office 365 software. Mean and standard deviations (SD) were used to report continuous data while categorical data were reported as frequencies and percentages. The Student's t-test and chi-squared test were applied to determine statistical significance. A p-value < 0.05 was considered statistically significant.

## Results

Demographic characteristics

A total of 40 patients were enrolled in the study. The median age of the cohort was four years (three months-‍16 years). The severity of the conditions was categorized as follows: - mild: 1 (2.5%), moderate: 10 (25.5%), and severe: 29 (72.5%). Inhibitors were not detected in 70% of cases, while 30.0% had inhibitors present. Of the 40 patients, two (5.0%) were on-demand treatment, while 38 (95%) were on prophylaxis. Furthermore, the average number of bleeding events one year before the study was 11.47 (Table 2).

**Table 2:** Baseline characteristics of the study population

Characteristics	Values
Gender, n (%)	
Male	40 (100)
Age range at diagnosis	Three months-‍16 years
Age group, n (%)	
<5 years	23 (57.5)
5-10 years	9 (22.5)
>10 years	8 (20)
Mean age at diagnosis	5.4 months
Median age at diagnosis	4 years
Severity, n (%)	
Mild	1 (2.5)
Moderate	10 (25.5)
Severe	29 (72.5)
Inhibitor status, n (%)	
With inhibitor	12 (30)
Without inhibitor	28 (70)
Prophylaxis/on-demand therapy, n (%)	
On prophylaxis	38 (95)
On-demand	2 (5)
No. of bleeding events	
Total no. of bleeding events 1 year prior	459
Average no. of bleeds 1 year before the study	11.47
Participants with target joints at baseline, n (%)	
Target joints present	30 (75)
No target joints	10 (25)

Comparison and analysis of HJHS at baseline and 24 weeks

**Figure 2:**
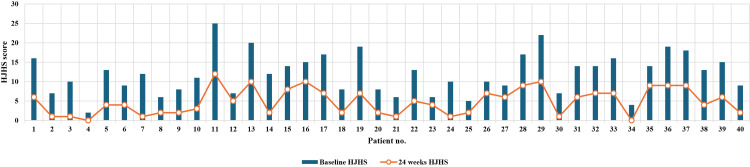
Individual comparison of HJHS at baseline and after 24 weeks HJHS: Hemophilia Joint Health Score

At baseline, none of the 40 participants had an HJHS score of 0; 18 participants (45%) had scores between 1 and 10, 20 (50%) participants had scores between 11 and 20, and two (5%) participants had scores between 21 and 30. The mean score was 12.0 ±5.23. After 24 weeks, two (5%) participants had a score of 0, 37 (92.5%) had scores between 1 and 10, and only one participant (2.5%) had a score between 11 and 20. The mean score decreased to 4.8 ±3.34 (Table 3). This change was statistically significant (p<0.001), indicating a substantial improvement in scores (Figure 2). There was a consistent reduction in HJHS scores over 24 weeks, indicating an overall improvement in joint health across participants. At 24 weeks, all 40 (100%) patients showed improvement in their HJHS with no deterioration or lack of improvement.

**Table 3:** Comparative analyses of different variables at baseline and 24 weeks after emicizumab prophylaxis EQ-5D-5L: European Quality of Life 5‐Dimensions 5‐levels; FISH: Functional Independence Score in Hemophilia; HJHS: Hemophilia Joint Health Score; SD: standard deviation; VAS: visual analog scale

	Baseline, mean ± SD	At 24 weeks, mean ± SD	P-value
HJHS score	12.0 ±5.23	4.8 ±3.34	<0.001
FISH score	27.5 ±2.11	30.6 ±1.32	<0.001
EQ-5D-5L score index	0.794 ±0.108	0.961 ±0.044	<0.001
VAS score	72.18 ±10.099	92.75 ±7.228	<0.001

Comparison and analysis of FISH at baseline and 24 weeks

**Figure 3:**
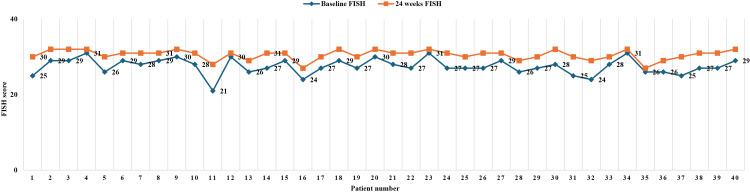
Individual comparison of FISH at baseline and after 24 weeks FISH: Functional Independence Score in Hemophilia

At baseline, 92.5% of patients had a FISH score between 24 and 32, and 7.5% had scores between 16 and 24 (Table 3). At 24 weeks, all patients (100%) had scores between 24 and 32. The mean FISH score increased from 27.5 ±2.11 at baseline to 30.6 ±1.32 at 24 weeks, which was statistically significant (p<0.001). The FISH score increased for all patients after 24 weeks, indicating an improvement or maintenance of functional independence during the study period (Figure 3). At 24 weeks, all 40 (100%) patients showed an improved FISH score, with no patients experiencing deterioration or no improvement.

Analysis of ABR, annual joint bleed rate, and annual target joint bleed rate at 24 weeks

**Figure 4:**
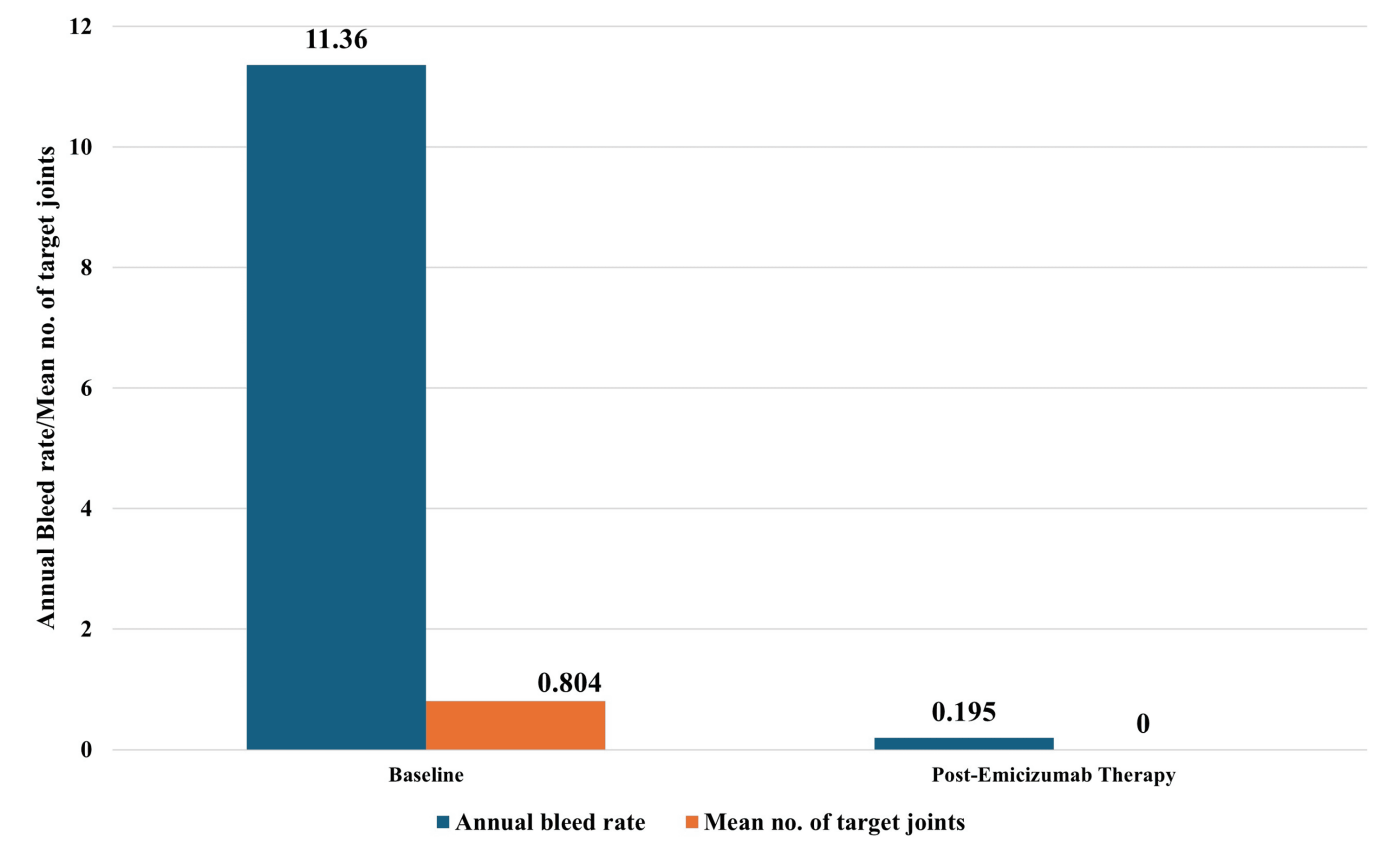
Comparative analyses of ABR and mean no. of target joints at baseline and after 24 weeks ABR: annual bleeding rate

**Figure 5:**
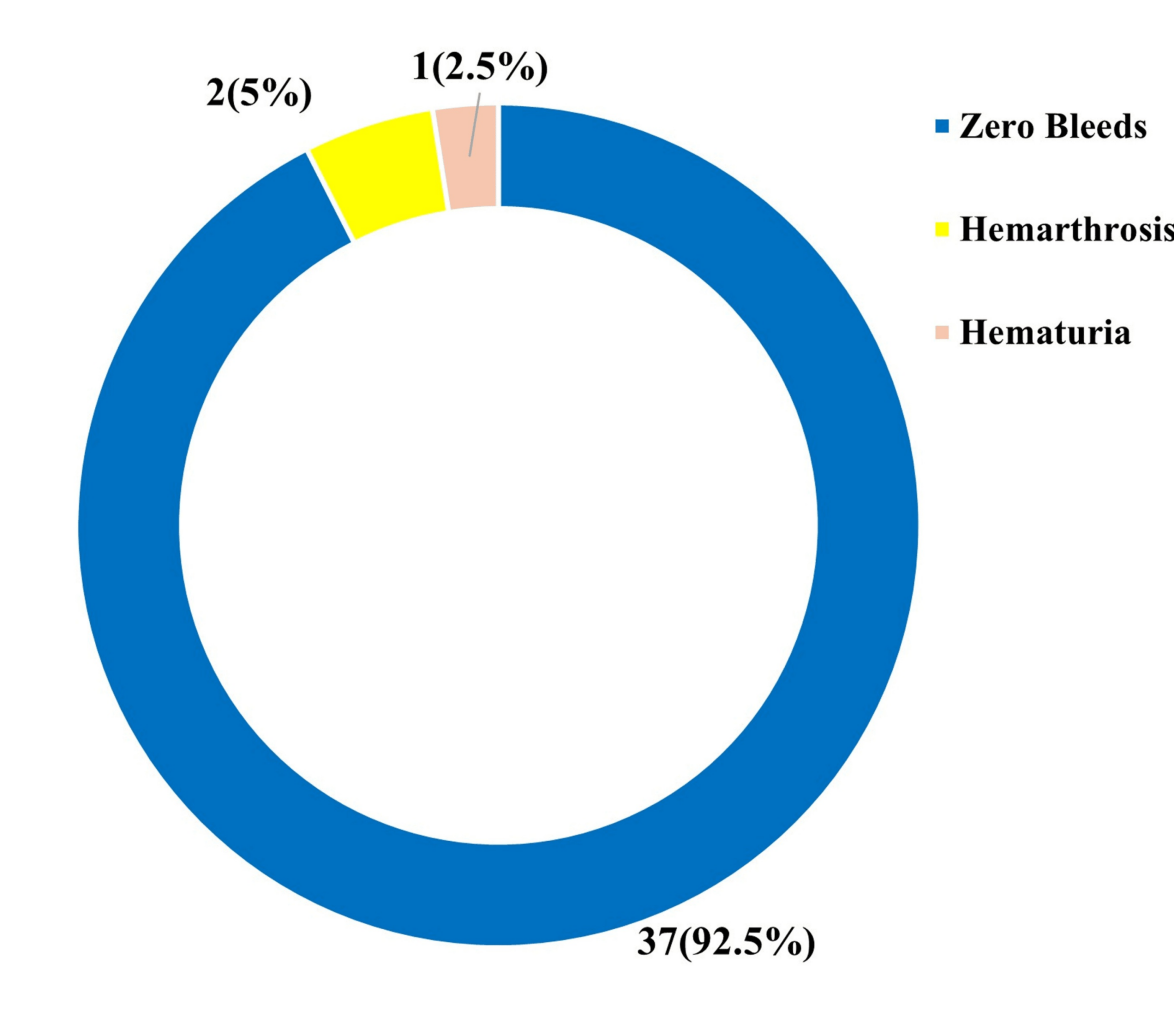
Incidence of bleeding episodes in patients after emicizumab prophylaxis

All patients showed improvement in both ABR and annual joint bleeds. Among the 40 patients, 30 had target joints, and in all of these 30 patients, the number of target joints reduced to zero following emicizumab therapy. Specifically, the ABR improved significantly from a baseline of 11.36 to 0.804, and the number of target joints decreased from 0.195 to 0.000 (Figure 4). A total of 37 patients (92.5%) reported no bleeds following emicizumab prophylaxis. Of the remaining three patients, one experienced hematuria (2.5%), and two had hemarthrosis (5%) (Figure 5).

Improvement in HRQoL

**Figure 6:**
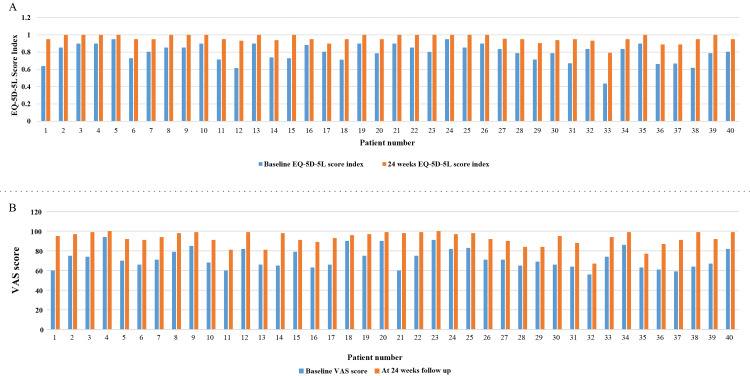
Comparison of individual EQ-5D-5L score index (A) and VAS score (B) between baseline and the first visit after emicizumab therapy EQ-5D-5L: European Quality of Life 5-Dimensions 5-Levels; VAS: visual analog scale

At 24 weeks, all 40 (100%) patients demonstrated an improved EQ-5D-5L score, with no patients experiencing deterioration or lack of improvement. The study results show significant improvements from baseline to the first follow-up. The EQ-5D-5L score index increased from a mean of 0.79 (SD: 0.108) to 0.96 (SD: 0.044) (Figure 6A), and the visual analog scale (VAS) score rose from a mean of 72.18 (SD: 10.099) to 92.75 (SD: 7.23) (Figure 6B). Both changes were associated with a p-value of <0.001, indicating statistically significant improvements in QoL and perceived health status (Table 3; Figures 6A, 6B). In all patients, there was a general increase in the EQ-5D-5L score index, suggesting improved QoL, and an increase in VAS scores, indicating reduced pain levels, over the 24-week follow-up period.

Adherence

All patients exhibited 100% adherence to emicizumab treatment, with no missed doses and consistent attendance at monthly follow-ups.

## Discussion

Emicizumab is the first nonfactor replacement therapy for HA, and it has demonstrated significant effectiveness in meeting treatment needs [10]. In this hospital-based, prospective, noninterventional observational study involving 40 patients with congenital HA, significant improvements were observed across all measured endpoints following treatment with emicizumab. Furthermore, all patients adhered fully to the emicizumab treatment regimen, with no missed doses or follow-up appointments. This high level of adherence highlights the feasibility and acceptability of emicizumab as a treatment option in real-world clinical settings.

The HJHS showed a marked improvement, decreasing from a mean of 12.0 ±5.23 at baseline to 4.8 ±3.34 at 24 weeks (p<0.001). In parallel, the FISH increased from 27.5 ±2.11 to 30.6 ±1.32. These improvements were accompanied by substantial reductions in the ABR, annual joint bleed rate, and annual target joint bleed rate at 24 weeks. Furthermore, the study demonstrated significant enhancements in the EQ-5D-5L score index and VAS scores from baseline to the first visit. The EQ-5D-5L score index increased from 0.794 to 0.961, and the VAS scores rose from 72.18 to 92.75, both with p-values of <0.001. These results suggest a positive impact of emicizumab on HRQoL and perceived health status.

Our study findings align with global research on the efficacy and safety of emicizumab [22-24], while also highlighting unique insights specific to the population in northeastern India [10,22-24]. The preliminary phase I study done by Shima et al. [25] demonstrated the therapeutic efficacy of emicizumab in preventing bleeding episodes in PwHA. This proof-of-concept study showed that weekly subcutaneous doses of 0.3, 1, or 3 mg/kg led to zero treated bleeds in three, five, and five patients, respectively, after 12 weeks. Median ABRs were 4.4, 0.0, and 0.0, respectively. These low ABRs were sustained in a long-term phase I/II extension study, informing the dosing regimens for subsequent phase III trials [10,25]. Furthermore, various studies investigating the clinical efficacy of emicizumab, including HAVEN 1, HAVEN 2, and HAVEN 3, have reported significant reductions in ABR with emicizumab compared with other treatments: 87% in HAVEN 1 [22], 99% in HAVEN 2 [23], and 96%-97% in HAVEN 3 [24].

Further endorsing these findings, HAVEN 4 demonstrated that monthly dosing of emicizumab (6 mg/kg) effectively controlled bleeding in patients with severe HA or HA with inhibitors, resulting in an ABR of 2.4, with 56.1% of patients experiencing no treated bleeds [26]. HAVEN 5 showed that weekly or monthly emicizumab dosing reduced ABRs by 96% compared with no prophylaxis, with no thrombotic events or thrombotic microangiopathies (TMAs) reported [27]. In a real-world study from China by Liu et al., ABR decreased from 4 to 0.5 in pediatric patients with severe or moderate HA after switching to emicizumab, with 46% experiencing zero bleeds [28].

Moreover, HAVEN 6 reported that emicizumab significantly reduced ABR in patients with mild or moderate HA, with improved target joint issues and a favorable benefit-risk profile [29]. The HAVEN 7 study further showed that emicizumab administered from birth in infants with severe HA led to an ABR of 0.4 and no significant AEs, emphasizing its potential to prevent joint bleeds and intracranial hemorrhage [30]. Other real-world studies, such as HOHOEMI and STASEY, also support the safety and efficacy of emicizumab in diverse patient populations [31,32]. A recent Indian study by Radhakrishnan et al. showed a decrease in average bleed frequency from 2-3.5 per month to zero after starting emicizumab, with improvements in HRQoL [18]. They reported improvement in the EQ-5D-5L and VAS scores for all patients at 24 weeks, reflecting a positive impact on HRQoL. The HAVEN studies found that emicizumab prophylaxis led to overall improvements in HRQoL over 25 weeks in adolescents and adults with HA, both with and without FVIII inhibitors [8,27,29].

Similar results were observed in a recent retrospective study of 38 patients over 18 years receiving emicizumab, with evaluations conducted before and one year after treatment. Health, pain, bleeding, and quality of life were evaluated using various scales, and treatment satisfaction was reported. Results showed significant improvement in general health (EQ-5D-3L score: 69.6 ±19.4, p=0.0023), decreased pain intensity, reduced bleeding frequency, and high satisfaction (9.1/10). Emicizumab therapy led to improved health outcomes and high patient satisfaction [33].

Additionally, in a pooled analysis of 176 PwHA, aged 18+ years, on emicizumab prophylaxis, significant HRQoL improvements were found. The mean Haem-A-QoL Physical Health (PH) and Total Score (TS) increased by 12.0 and 8.6 points, respectively, from baseline to week 73, with 54% of patients reporting a clinically meaningful PH improvement (≥10 points). However, no change was observed on the EQ-5D-5L [8]. This discrepancy could be attributed to differences in baseline patient profiles, healthcare settings, or cultural perceptions of health and QoL. Similarly, another analysis aimed to determine clinically important responder (CIR) thresholds for three domains in adults with HA treated with emicizumab prophylaxis in the HAVEN 1, 3, and 4 studies. Using an anchor-based methodology with EQ-5D-5L as the anchor and distribution-based methods, CIR thresholds were calculated for improvements from baseline to 24 weeks. Concordance was found between Haem-A-QoL and EQ-‍5D-‍5L in patterns of improvement and deterioration [34].

Similar results were observed in a recent cohort study from Canada by Blydt-Hansen et al., where EQ-5D-5L index utility scores improved at both three and six months after initiating emicizumab. The minimal clinically important difference was achieved at three months for usual activities and anxiety/depression by one person and in all domains by at least one person at six months. VAS scores indicated a consistent perceived improvement by 42% at three months and by most participants at six months [35]. The findings in children align with those observed in adults. In a recent study involving 37 children with severe HA receiving emicizumab prophylaxis, the median pediatric hemophilia activities score improved from 57.6 to 76 (p<0.001), indicating increased physical activity. QoL measured by EQ-5D for youth showed significant improvement, with a higher proportion of patients reporting no problems in all dimensions (p<0.05). The VAS score increased from 67 to 76 [36].

Additionally, the 100% adherence rate in this study cohort highlights the feasibility and acceptability of emicizumab in resource-limited settings. While emicizumab has revolutionized hemophilia care globally, studies from India remain limited. This study addresses this gap by offering real-world data from northeastern India, where centralized care and limited access to advanced treatments pose challenges. The subcutaneous administration of emicizumab reduces hospital visits, benefiting patients in remote areas. Overall, emicizumab appears to be effective in improving joint health, functional activity, and QoL in patients with congenital HA, with significant clinical benefits observed within the first 24 weeks of treatment and positive trends expected to continue. Further research with larger, multicenter cohorts is needed to validate these findings and assess long-term outcomes, to promote broader adoption in resource-constrained settings. Further long-term data will provide additional insights into the sustained efficacy and safety of this therapy.

Limitations

This study has several limitations. The relatively small sample size and the single-center design may restrict the generalizability of the results. The observational nature of the study, which did not include a control group, means that the improvements observed could be attributed solely to emicizumab therapy. Hence, we recommend larger multicenter randomized controlled trials with extended follow-up to validate our findings.

## Conclusions

Our results suggest that emicizumab significantly enhances joint health, functional independence, and overall QoL in patients with congenital HA. Its subcutaneous administration, long-lasting effects, and good safety profile make it a strong alternative to traditional FVIII replacement therapy. The substantial improvements observed within the first 24 weeks of treatment underscore its potential as an effective long-term therapy for both pediatric and adult populations with HA. These findings indicate a critical need to integrate emicizumab into standard care protocols for patients with HA to optimize clinical outcomes. Moreover, this study emphasizes the importance of expanding access to advanced treatments such as emicizumab, particularly in resource-constrained regions. Establishing specialized diagnostic and therapeutic facilities in these areas could ensure that more patients benefit from emicizumab's advantages, such as reduced bleeding rates and improved QoL while minimizing the overall treatment burden. Overall, the study highlights the potential of emicizumab to transform HA management and significantly improve patient outcomes across diverse populations. However, the small sample size, single-center design, and absence of a control group limit the findings, necessitating larger multicenter randomized trials with longer follow-ups to validate the results.

## Figures and Tables

**Figure 7:**
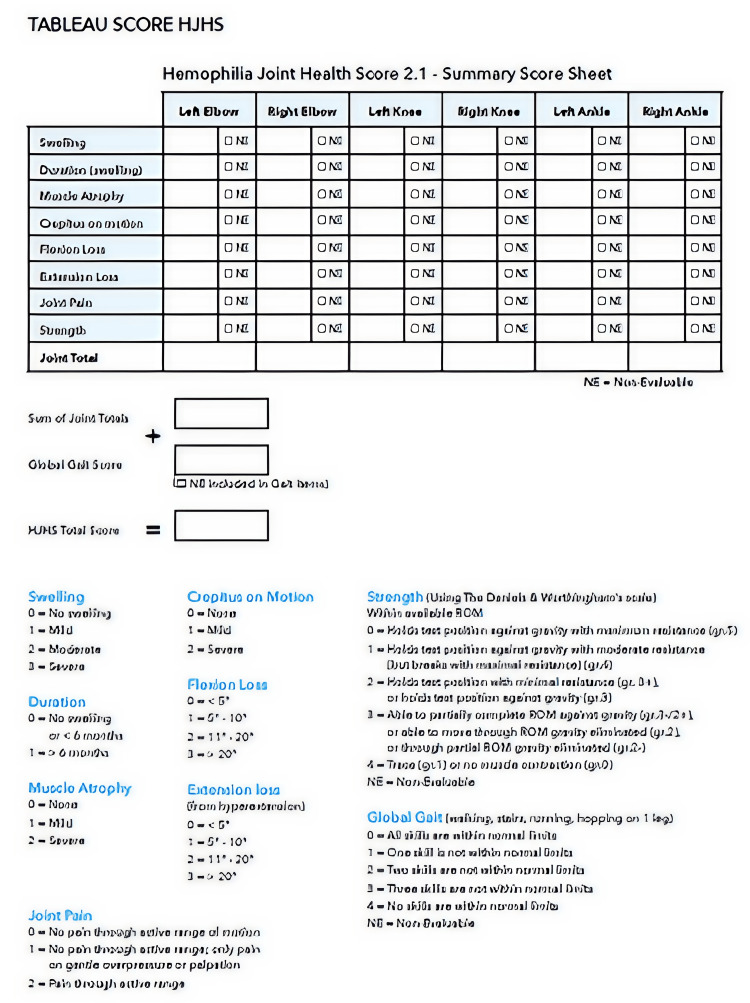
HJHS questionnaire https://www.hemophilink.fr/wp-content/uploads/2019/01/Hemophilia-joint-health-score-HJHS.pdf HJHS: Hemophilia Joint Health Score

**Figure 8:**
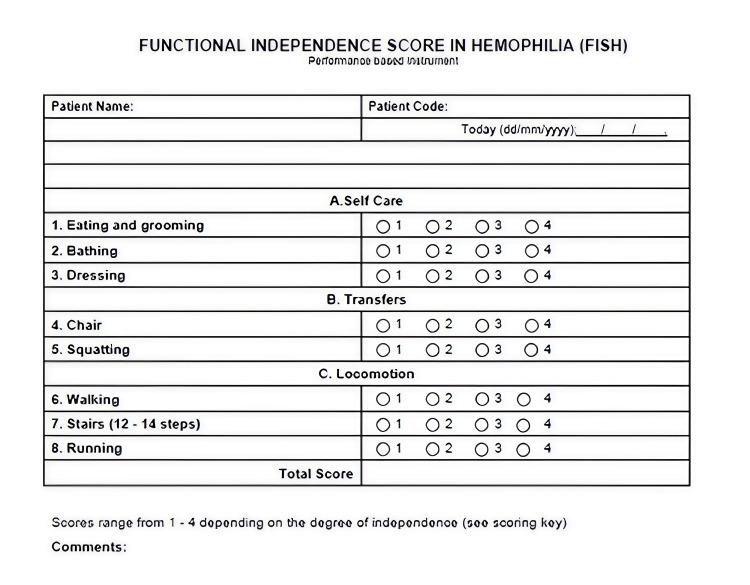
FISH questionnaire https://elearning.wfh.org/resource/functional-independence-score-in-hemophilia-fish/ FISH: Functional Independence Score in Hemophilia

**Figure 9:**
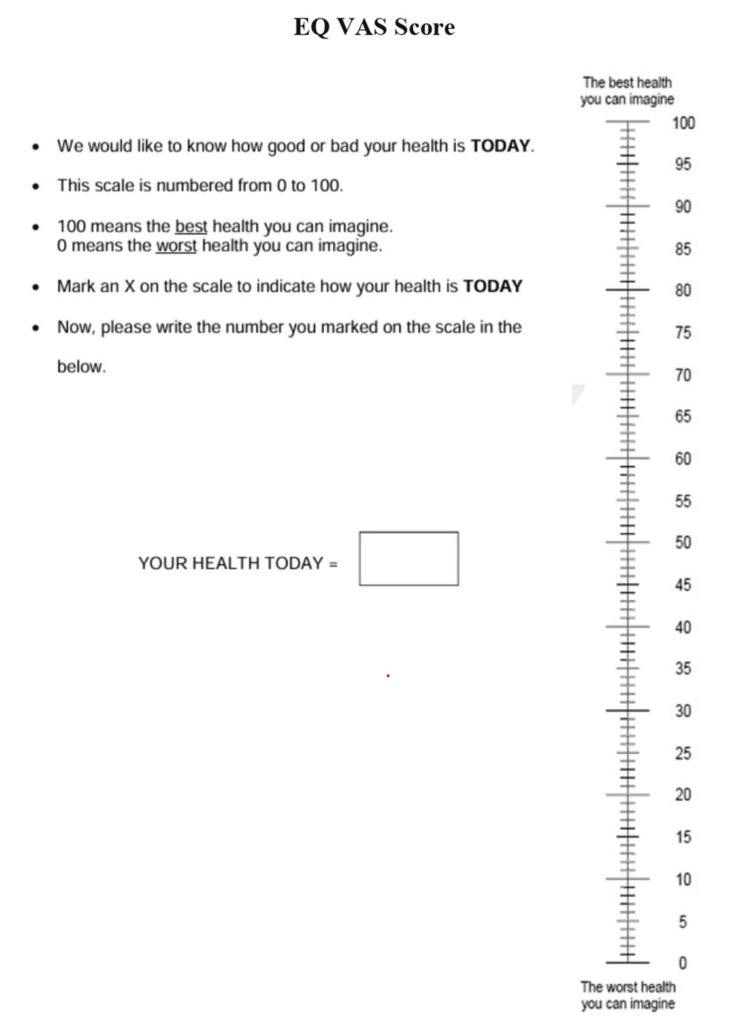
EQ VAS score questionnaire https://www.sciencedirect.com/science/article/pii/S1279770723010667 EQ-VAS: European Quality Visual Analog Scale

## Appendices

**Table 4:** EQ-5D-5L questionnaire https://euroqol.org/information-and-support/euroqol-instruments/eq-5d-5l/ EQ-5D-5L: European Quality of Life 5‐Dimensions 5‐levels

Under each heading, please tick the ONE box that best describes your health TODAY	
	Mobility
1	I have no problems in walking about
2	I have slight problems in walking about
3	I have moderate problems in walking about
4	I have severe problems in walking about
5	I am unable to walk about
	Self-care
1	I have no problems washing or dressing myself
2	I have slight problems washing or dressing myself
3	I have moderate problems washing or dressing myself
4	I have severe problems washing or dressing myself
5	I am unable to wash or dress myself
	Usual activities (e.g. work, study, housework, family or leisure activities)
1	I have no problems doing my usual activities
2	I have slight problems doing my usual activities
3	I have moderate problems doing my usual activities
4	I have severe problems doing my usual activities
5	I am unable to do my usual activities
	Pain/Discomfort
1	I have no pain or discomfort
2	I have slight pain or discomfort
3	I have moderate pain or discomfort
4	I have severe pain or discomfort
5	I have extreme pain or discomfort
	Anxiety/Depression
1	I am not anxious or depressed
2	I am slightly anxious or depressed
3	I am moderately anxious or depressed
4	I am severely anxious or depressed
5	I am extremely anxious or depressed
